# Risperidone-Induced Nasal Congestion and Increased Urinary Frequency

**DOI:** 10.7759/cureus.113937

**Published:** 2026-08-04

**Authors:** Hessa Abu Baker, Bushra Alnuaimi, Ali Alkot

**Affiliations:** 1 Medicine, Gulf Medical University, Ajman, ARE; 2 Internal Medicine, Gulf Medical University, Ajman, ARE; 3 Psychiatry, Al Amal Psychiatric Hospital, Dubai, ARE

**Keywords:** antipsychotic, increased urinary frequency, nasal congestion, risperidone, side effect

## Abstract

Risperidone is a second-generation antipsychotic that is widely used in the treatment of psychotic disorders. It is generally well tolerated, although uncommon adverse effects have been reported. Nasal congestion and urinary frequency have only rarely been described in association with risperidone treatment. A 27-year-old male with delusional disorder developed nasal congestion and urinary frequency after starting risperidone. He underwent evaluation by both family medicine and otolaryngology (ENT), and no alternative cause of his symptoms was identified. Despite treatment with antihistamines, intranasal budesonide, and xylometazoline, his symptoms persisted. The symptoms resolved when he stopped taking risperidone for two days and returned when the medication was restarted. Risperidone was subsequently discontinued and replaced with aripiprazole. Following the switch, the patient’s symptoms resolved completely. This case suggests a probable association between risperidone and the development of nasal congestion and urinary frequency. Awareness of these uncommon adverse effects may help clinicians identify medication-related symptoms, avoid unnecessary investigations, and improve treatment adherence.

## Introduction

Risperidone is a second-generation antipsychotic commonly used in the treatment of schizophrenia, bipolar disorder, delusional disorder, and other psychotic conditions [1]. Typical maintenance doses in adults generally range from 2 mg to 6 mg daily. Common adverse effects include somnolence, weight gain, dizziness, extrapyramidal symptoms, akathisia, tremor, and anxiety [2]. Although nasal congestion and urinary frequency are generally considered nonspecific symptoms with many possible causes, they may represent uncommon adverse effects of risperidone that can be overlooked in routine clinical practice. Recognition of these reactions is important because they may lead to unnecessary investigations, reduced medication adherence, or premature discontinuation of treatment. Reporting additional cases contributes to the limited literature and increases awareness among clinicians regarding these rare adverse effects.

We report the case of a patient who developed nasal congestion and urinary frequency during risperidone treatment. The symptoms resolved after discontinuation of the medication and recurred when treatment was resumed. Nasal congestion and urinary symptoms have been reported infrequently during risperidone treatment and have been described mainly in isolated case reports rather than in large clinical studies [3]. The therapeutic effects of risperidone are thought to result primarily from antagonism of dopamine D2 and serotonin 5-HT2A receptors. Risperidone also acts on histamine H1 receptors and α1- and α2-adrenergic receptors [4]. Its action on peripheral α1-adrenergic receptors has been proposed as a possible mechanism for some uncommon adverse effects. Blockade of α1-adrenergic receptors in the nasal mucosa may lead to vasodilation and mucosal swelling, resulting in nasal obstruction [3,4]. Similar receptor activity has also been proposed as a possible explanation for certain urinary symptoms reported during treatment.

## Case presentation

A 27-year-old male with a known diagnosis of delusional disorder attended an outpatient psychiatric clinic for follow-up. He complained of nasal congestion and increased urinary frequency. There was no significant medical, surgical, social, or family history, and he had no known allergies. Vital signs were stable, and physical examination revealed no abnormal findings. The patient started risperidone 2 mg twice daily in May 2024. Approximately one week later, he reported dizziness and difficulty concentrating. The dosage was changed to 4 mg at night. At a follow-up visit in June 2024, he continued to report lightheadedness and difficulty concentrating, and the dose was reduced to 2 mg daily.

During a follow-up appointment in August 2024, the patient reported persistent nasal congestion and increased urinary frequency. He was referred to both family medicine and otolaryngology (ENT) for further assessment. At a subsequent psychiatric follow-up in November 2024, the patient reported that the ENT evaluation had excluded structural nasal abnormalities, nasal polyps, sinusitis, and allergic rhinitis. He had also been assessed by family medicine, where additional investigations were performed and reported as normal. Despite treatment with antihistamines, intranasal budesonide, and xylometazoline, his symptoms persisted. The patient also reported that he had run out of risperidone and missed two days of treatment. During this period, both the nasal congestion and urinary frequency resolved. After restarting risperidone, the symptoms returned. This raised suspicion that the symptoms might be related to the medication.

In December 2024, risperidone was reduced to 1 mg daily for two weeks and then discontinued. Aripiprazole was started at 5 mg daily and increased to 10 mg daily after two weeks. At follow-up one month later, the patient reported complete resolution of both symptoms and remained well on aripiprazole.

The timeline of clinical events is summarized in Table 1.

**Table 1:** Timeline of clinical events.

Day	Clinical course
Day 0	Risperidone initiated at 2 mg twice daily.
Day 7	Dizziness and poor concentration developed. Dose changed to 4 mg nightly.
Day 30	Symptoms persisted. Dose reduced to 2 mg daily.
Day 90	Patient reported increased urinary frequency and nasal congestion. Referred to otolaryngology (ENT) and family medicine.
Day 180	Symptoms resolved after missing risperidone for two days and recurred after restarting it.
Day 210	Risperidone tapered and discontinued. Aripiprazole 5 mg/day initiated.
Day 224	Aripiprazole increased to 10 mg/day.
Day 254	All adverse effects resolved. The patient was satisfied with the new medication.

## Discussion

This case describes the development of nasal congestion and urinary frequency in a patient taking risperidone. No alternative cause was identified despite assessment by otolaryngology (ENT) and family medicine. The symptoms resolved when risperidone was stopped and returned when it was restarted, suggesting a probable association with the medication. Risperidone is generally well tolerated; however, several adverse effects have been well documented. Common adverse effects include somnolence, weight gain, dizziness, hyperprolactinemia, extrapyramidal symptoms, akathisia, tremor, parkinsonism, orthostatic hypotension, and metabolic disturbances. Less common adverse effects include urinary disturbances, nasal congestion, sexual dysfunction, and cardiac conduction abnormalities. Compared with these well-recognized adverse effects, nasal congestion and urinary frequency remain rarely reported, making the present case clinically relevant.

Risperidone has affinity for dopamine D2, serotonin 5-HT2A, histamine H1, and α1- and α2-adrenergic receptors [1]. Although the exact mechanism responsible for nasal congestion is not known, α1-adrenergic receptor blockade has been proposed as a possible explanation. Reduced sympathetic vasoconstriction within the nasal mucosa may lead to vasodilation, mucosal swelling, and nasal obstruction. Reports of risperidone-associated nasal congestion are limited. A case report described a patient who developed persistent nasal obstruction after treatment with risperidone at a dose of 6 mg/day. The patient had no prior history of nasal disease, and evaluation by an otolaryngologist, including imaging, excluded structural abnormalities such as septal deviation, nasal polyps, and sinus disease. As no alternative cause for the symptoms was identified, risperidone-induced nasal obstruction was suspected. Following discontinuation of risperidone and a switch to an alternative antipsychotic, the patient’s nasal symptoms resolved completely [5]. Nasal congestion has also been reported with other atypical antipsychotics that possess α1-adrenergic receptor antagonistic properties. Jensen and Andersen reported the case of a 17-year-old patient with schizophrenia who developed persistent, severe nasal congestion after initiating quetiapine. The symptoms progressively worsened despite treatment with intranasal corticosteroids and antihistamines. Evaluation by an otolaryngologist excluded allergic and significant structural causes; however, the nasal obstruction was severe enough that surgical intervention was being considered. Quetiapine was subsequently discontinued and replaced with lurasidone, resulting in a rapid and complete resolution of the nasal congestion [6].

Urinary adverse effects associated with risperidone have been described less frequently than metabolic or neurological adverse effects. The mechanism remains unclear. One proposed explanation is that α1-adrenergic receptor blockade may alter normal lower urinary tract function and contribute to symptoms such as urinary frequency, urgency, or incontinence. A recent case report described a 36-year-old male with schizoaffective disorder, depressive type, who was started on risperidone after years of poor response and noncompliance with other psychotropic medications. Following initiation of risperidone, he showed marked improvement in his symptoms, and his family reported significant clinical stabilization compared with previous years. However, he subsequently developed urinary frequency, nocturnal enuresis, and urinary incontinence, which progressively worsened during treatment. Medical investigations ruled out common urological and metabolic causes, supporting a possible association with risperidone. Because of the substantial psychiatric benefit, risperidone was continued, and the urinary symptoms were managed symptomatically. When oxybutynin, an antimuscarinic medication, was started, the patient’s psychiatric stability was preserved, and the urinary symptoms resolved completely [7].

The Naranjo Adverse Drug Reaction Probability Scale was used to assess causality [8]. The calculated score was 8, indicating a probable adverse drug reaction. The score was supported by previous reports of similar reactions, the temporal relationship between drug initiation and symptom onset, improvement after discontinuation, recurrence after rechallenge, and the absence of an alternative explanation. In addition to the Naranjo Adverse Drug Reaction Probability Scale, the Schumock and Thornton Preventability Scale was applied to assess whether the adverse drug reaction could have been prevented [9]. The reaction was classified as not preventable, as risperidone was prescribed at an appropriate dose for an accepted indication, there was no documented history of similar reactions or contraindications, no clinically significant drug interactions were identified, and appropriate clinical evaluation was performed following symptom onset.

## Conclusions

This case describes nasal congestion and urinary frequency that developed during risperidone treatment and resolved after the medication was discontinued. The recurrence of symptoms after the patient restarted risperidone further supports a probable association. When patients receiving risperidone present with unexplained nasal congestion or urinary symptoms, the medication should be considered a possible contributing factor, particularly when routine investigations do not identify another cause. Although the findings support a probable association, this remains a single case report and cannot establish a definite causal relationship between risperidone and these symptoms. Additional case reports and pharmacovigilance studies are needed to better understand these uncommon adverse effects and their underlying mechanisms.
